# What Do We Know About Bacterial Infections in Hidradenitis Suppurativa?—A Narrative Review

**DOI:** 10.3390/antibiotics14020142

**Published:** 2025-02-01

**Authors:** Zuzanna Świerczewska, Wioletta Barańska-Rybak

**Affiliations:** Department of Dermatology, Venereology, and Allergology, Faculty of Medicine, Medical University of Gdańsk, Smoluchowskiego 17, 80-214 Gdańsk, Poland; zuzanna.swierczewska@gumed.edu.pl

**Keywords:** hidradenitis suppurativa, acne inversa, infections, bacteria

## Abstract

**Background/Objectives**: Hidradenitis suppurativa is an inflammatory skin condition of the pilosebaceous unit of a chronic, painful, and progressive nature. It affects intertriginous parts of the body, including the axillae, groin, submammary region, and anogenital region. The risk of infection in HS patients is not well understood. Thus, presenting the most recent findings in the study of bacterial infections in relation to hidradenitis suppurativa was the objective of this review. **Methods**: The presented article is a narrative review. The PubMed and Scopus databases were searched for articles applicable to this review. All types of study design were included in this review. **Results**: Among reported infections in patients with HS, Fournier’s gangrene, osteomyelitis, *Clostridium difficile* infection, and biofilm were significant. Attention should also be paid to post-procedural infections. **Conclusions**: A wide range of bacterial infections, from localized purulent infections to serious systemic consequences, can affect patients with HS. Comorbid diseases like diabetes mellitus and obesity change the cutaneous microbiota and produce a pro-inflammatory systemic milieu, which makes the disease more severe and makes HS patients more susceptible to infections. Additionally, those with untreated or unmanaged HS are more likely to experience infectious complications.

## 1. Introduction

Hidradenitis suppurativa (HS; acne inversa) is a chronic, painful, and progressive inflammatory skin disease of the pilosebaceous unit, affecting intertriginous areas of the body such as the axillae, groin, submammary or anogenital region. The diagnosis is made based on a clinical picture of typical HS lesions such as nodules, abscesses, fistulas, and scars [1,2]. The severity of HS and disease burden can be assessed with the use of several tools including, for instance, the Hurley scoring system, international hidradenitis suppurativa severity score system (IHS4), hidradenitis suppurativa physician’s global assessment (HS-PGA) scale, hidradenitis suppurativa clinical response (HiSCR), and dermatology life quality index (DLQI). HS typically begins in early adulthood and affects both men and women, with a ratio of 1:3. Although rare, the first manifestations can also emerge in childhood.

The pathogenesis of HS is considered as multifactorial, with components such as genetics, lifestyle, environment, hormones, and microbiota all taking part in this process. While HS is not considered an infectious skin condition, immunological activation may result from bacterial transmission in the intertriginous regions, where skin folds often contact and rub. Pro-inflammatory cytokines including TNF, IL-1β, and IL-17 are secreted by both innate and adaptive immune system cells, activating tissue cells and causing immune cell infiltration and further inflammation [1,3]. This ultimately leads to pus production, tissue degradation, and scar development in HS patients.

There is little information on the risk of infection in patients with HS. In this review, we aimed to present the latest reports in the field of research on bacterial infections in terms of hidradenitis suppurativa.

## 2. Pathogenesis and Role of Bacteria

**Figure 1:**
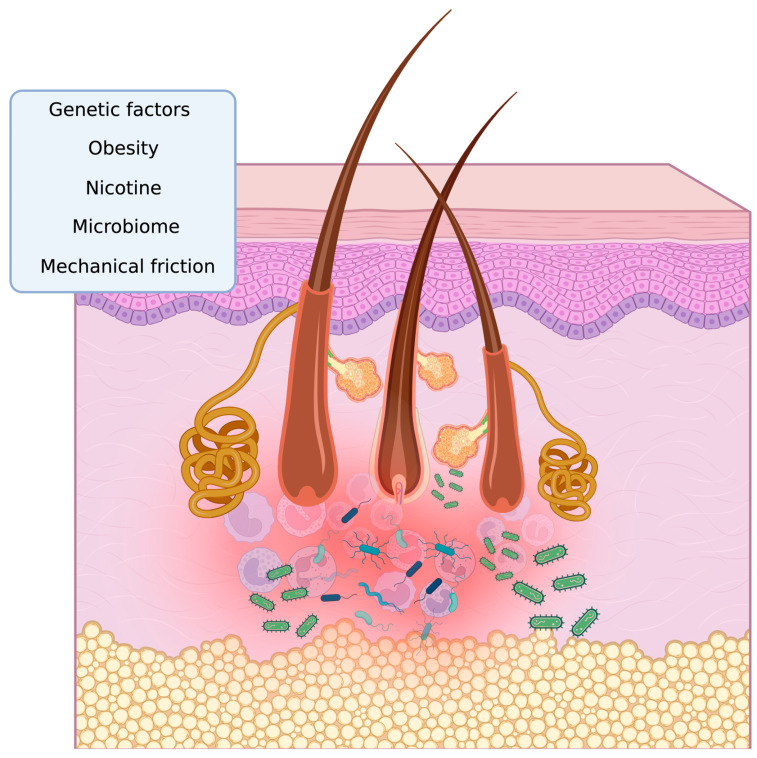
The factors influencing the pathogenesis of HS: a simplified overview. Genetic factors, obesity, nicotine, mechanical friction, microbial dysbiosis, and biofilm stimulate the inflammatory state in HS. Immune cells such as neutrophils, macrophages, dendritic cells, and keratinocytes act as key mediators, releasing pro-inflammatory cytokines and sustaining the inflammatory environment. In obese individuals, the aggravation of HS is associated with mechanical stress, which increases follicular occlusion and follicular rupture. Studies also show that up to 40% of HS patients have a family history of the condition, highlighting the importance of genetic factors. Altogether, these factors lead to a continuous vicious cycle of chronic inflammation and further exacerbate the disease. Image created with BioRender.com.

Bacterial colonization and infection are increasingly recognized as critical contributors to HS progression and chronicity. Dysbiosis of the skin microbiome in HS lesions reveals an over-representation of anaerobic species, including Prevotella, Peptoniphilus, and *Porphyromonas* spp., and a loss of skin commensal species, such as Cutibacterium [4]. Through a variety of pathogenic routes, these microbes may contribute to the pathophysiology of HS. The rupture of hair follicles in HS leads to the release of keratin and other debris into the dermis, creating a nidus for bacterial colonization [5]. This results in the secretion of pro-inflammatory cytokines such as tumor necrosis factor-alpha (TNF-α), interleukin (IL)-1β, IL-6, and IL-17, which contribute to the chronic inflammatory state of HS. (Figure 1).

Furthermore, biofilm and its function in the etiology of HS also deserve discussion in this review. Studies have shown that HS is a disease marked by the production of biofilms, which might be a trigger for the organism–host response [2,6,7]. Remarkably, as compared to healthy controls and HS patients without biofilm, patients with HS and identified biofilms have been found to have an increased concentration of CD4+ cells. Therefore, it has been proposed that biofilm promotes the formation of regulatory T cells [8].

Interestingly, studies have indicated that superantigens produced by Staphylococcus aureus can activate T cells, leading to autoreactive immune responses [9,10]. This mechanism may contribute to the chronic and systemic nature of certain diseases. Although the exact timing of bacterial colonization and HS onset is unclear, evidence suggests that immune dysregulation and microbial imbalance may interact in a way that worsens disease severity. The factors influencing the pathogenesis of HS are shown in Figure 1.

## 3. Risk of Certain Infections Among HS Individuals

Upon the removal of duplicates, 6308 abstracts and titles were assessed. Our search identified 23 records eligible for this study after examining their full texts. The data found revealed that individuals with HS are more prone to developing certain infections. A summary of reported cases of bacterial infections in HS patients is presented in Table 1.

### 3.1. Risk of Infection in Patients with HS on Biologics or Other Immunomodulators

Biologic drugs have recently emerged as a viable therapy option for HS, with researchers investigating their ability to find therapeutic targets such as interleukins 1 and 17. Anti-TNF drugs, IL-12/23 inhibitors, and anti-IL-17 medications have shown promising success in decreasing HS symptoms and improving patients’ quality of life. Infections associated with biologic therapy are more likely to be severe or even fatal [17]. Patients with HS are believed to be prone to infectious problems due to the dysregulation of the immune system, bacterial colonization, skin barrier disturbances, and the presence of comorbidities [18]. Thus, it is important to carefully consider the risks and benefits of therapies.

However, a meta-analysis from 2024 found that the risk of infection among HS patients treated with biologics was at 24.21%, which was not significantly greater than that of placebo [19]. While severe and opportunistic infections seem to be quite uncommon, the data show that therapy with these newer drugs does not significantly raise the risk of infectious repercussions. Nonetheless, when treating HS patients with biologics, clinicians must exercise caution, follow all advised primary preventive strategies, including immunization, and monitor patients for any indications of infection.

### 3.2. Risk of Infection in Patients with HS on Antibiotics (C. difficile Infection)

In HS, antibiotic monotherapy is frequently utilized as the first-line treatment. Previous studies have shown that the widespread use of antibiotics in the treatment of HS increases the risk of *C. difficile* infection (CDI) and increased mortality [20]. Yet, conflicting evidence is presented in recent publications. As rightly noted by Bessaleli et al., rifampicin in one of the treatment options for CDI [11]. Therefore, when used in combination with clindamycin, as it often is in the treatment of HS, it ought to decrease the risk of CDI development. Nonetheless, prior exposure to rifampicin and its prolonged use are believed to be the risk factors for rifampicin-resistant CDI; thus, clinicians must be aware of any determinants.

Sanghvi et al. tried to assess the risk of *C. difficile* infection among patients with HS [20]. The incidence of CDI in HS patients was 11% (*n* = 98), compared with 0.18% (*n* = 12 630) in non-HS patients. Noteworthy, in HS patients CDI was more likely among older patients, those with comorbid inflammatory bowel disease (IBD), and those who were exposed to more distinct antibiotics. The risk also increased with the number of hospitalizations. An increased risk particularly for quinolones and penicillins was discovered. Nevertheless, the risk of CDI did not appear to be raised by routinely used antibiotics in HS, such as clindamycin, cephalosporins, and tetracyclines. In order to guarantee a lower screening threshold in the event that symptoms appear, patients should be advised to monitor for potential CDI symptoms.

On the other hand, a study by Kwak et al. revealed no increased risk of CDI in HS patients receiving oral or IV clindamycin [21]. Nonetheless, the study was constrained by its small sample size and a retrospective approach.

### 3.3. Post-Procedural Infections and Wound Healing

In order to control symptoms, avoid complications, and enhance patients’ quality of life, surgical treatments for HS differ according to the severity and scope of the disease. Wide excision, laser treatments, deroofing, or incision and drainage are among the common procedures; however, they are not without ramifications. Factors such as the surgery location, degree of disease involvement, and presence of comorbidities all take part in potential complication occurrence. Moreover, it has been demonstrated that the diverse microbiome seen in HS lesions increases the probability of a heterogeneous bacterial infection [22,23]. However, data on post-procedural infections among patients with HS is limited.

A retrospective 16-year cohort study from 2024 enrolled 136 patients with HS who underwent complete excision [24]. In total, 284 complete excisions were identified, and complications arose in 35.9% (*n* = 102). Among those, post-procedural wound infections were identified in 12.7%. In another retrospective cohort study on 670 patients with HS, surgical site infections (SSIs) were 8.36 times more likely compared to a placebo group (*n* = 3350; *p* < 0.0001). [25] According to the above-mentioned study, incision and drainage were more common among HS patients suffering from SSIs (91.76% vs. 72.03%; *p* < 0.0001). Even after controlling for common HS comorbidities such as obesity, diabetes, or nicotinism, HS was still independently related to a higher risk of developing SSIs.

Interesting data were also provided by Gouzoulis et al. [23]. In their study, they investigated adverse events after total hip arthroplasty (THA) or total knee arthroplasty (TKA). Among the studied groups, 481 HS patients for THA and 290 HS patients for TKA were identified. In comparison to patients without HS, those with HS following THA and TKA generally had comparable short-term cumulative results and 5-year implant survival. Following THA, HS patients had increased odds of wound dehiscence (*p* = 0.002). Greater probability of surgical site infection (*p* = 0.006) following TKA was also recorded. Even after controlling for age, sex, and ECI, HS was linked to more wound-related problems in the 90 days after surgery in both procedures. Given the paucity of existing research on the subject, these findings might benefit both patients and surgeons.

The study by Poondru et al. highlights a significant gap in wound care education for HS patients, with 70% of surveyed dermatologists perceiving patients as poorly educated on wound care practices, and 90% emphasizing the need for additional educational efforts [26]. This underscores the critical importance of integrating patient-centered education into HS management to improve outcomes, reduce complications, and empower patients in their self-care practices.

### 3.4. HS and Biofilm Formation

Biofilms are colonies of microorganisms that adhere to a surface and play an important role in the persistence of bacterial infections [27]. It is believed that biofilms give bacteria a shelter and help them develop a high tolerance to antibiotics and host defense clearance. In clinical practice, chronic inflammation, resistance to antibiotic treatment, and poor wound healing are characteristics of biofilm-driven diseases. Research suggests that biofilm may also contribute to HS. Nevertheless, since biofilm is primarily detected in sinus tracts during more severe stages of the disease, it is likely that HS does not begin as a biofilm disease but rather develops into one.

According to Ring et al., chronic HS lesions demonstrate multiple characteristics that are consistent with a biofilm-driven disorder, including inflammation, an unpredictable response to antibiotics, and impaired healing [7]. Furthermore, chronic HS lesions frequently contain keratin debris and hair pieces, which act as foreign bodies, potentially promoting biofilm formation. In their study, Ring et al. obtained biopsies from lesional as well as perilesional skin from 42 HS patients suffering from chronic HS lesions. In 75% of the perilesional samples and 67% of the chronic lesion samples, biofilms were seen. Lesional skin had aggregates with a substantially larger mean diameter than perilesional skin (*p* = 0.01). Most of aggregates larger than 50 µm were found in the infundibulum (37%) or sinus tracts (63%). Moreover, active bacterial cells, linked to inflammation, were found in 73% of the sinus tract samples.

Interestingly, Ardon et al. took skin biopsies of 26 HS patients from active HS lesions (inflammatory nodules and/or sinuses) and non-involved skin [28]. In 62% of patients (*n* = 16), *Staphylococcus epidermidis* was cultured with as many as 27 different isolates identified. In vitro, 24 out of 27 isolates (89%) produced robust biofilms. Moreover, the strains from non-involved and lesional skin had different biofilm-forming abilities. Concerningly, clindamycin susceptibility in 24 strains ranged from moderate to resistant. The antibiotic that eradicated biofilm with the greatest efficiency was rifampicin (*p* < 0.05).

Moreover, in a case report by Kathju et al., a confocal microscopy analysis of tissue samples found clusters of bacteria adhered to the sinus luminal surfaces in a 47-year-old female patient diagnosed with HS in her buttocks [6]. Furthermore, antibiotics were unable to fully cure the disease. Taken all together, the authors concluded the case to fall into category of a biofilm disease.

### 3.5. Risk of Sepsis in HS Patients

According to the latest literature reports, HS is commonly mistaken for an infection, and as a result, patients are treated for recurrent bacterial abscesses or admitted for sepsis. However, as a non-infectious disease, HS itself should not cause sepsis. A systematic review from 2024 revealed no case of sepsis in patients with HS that could be accredited to disease exacerbation or solely to HS [29]. Out of the analyzed cases, three were suggestive of disease aggravation with symptoms of SIRS (systemic inflammatory response syndrome), all with scantily controlled HS. According to the authors, patients with severe exacerbations of HS develop fever and SIRS, rather than sepsis. Nonetheless, in any patient with the presence of symptoms suggesting organ dysfunction or symptoms indicating infection, further assessment is mandatory.

On the other hand, an observational study by Ehizogieet al. analyzed data from the National Inpatient Sample (NIS) to assess the outcomes of patients with HS and a sepsis diagnosis compared with patients with sepsis only [30]. The search revealed 7 144 018 hospitalizations with a diagnosis of sepsis, with 8240 occurring in patients with HS. Compared with patients without HS, patients with the disease were younger, and mainly female. Moreover, sepsis hospitalizations in patients with HS had a lower inpatient mortality (1.6% vs. 11.6%, *p* = 0.002) and shorter length of stay (8.1 vs. 8.4 days, *p* < 0.0001). Unspecified organism was the most frequent cause of hospitalization overall and of in-hospital fatalities in both HS and non-HS sepsis hospitalizations. According to the NIS database, the death rate for sepsis in HS is 1.6%, or less than one in sixty, although the mortality rate for sepsis in patients without HS is greater than one in nine. This implies an overdiagnosis of sepsis in HS patients. The authors claim that all 27 of the database’s typical patients who died had significant comorbidities that helped to explain their deaths and increased risk of sepsis.

Similar conclusions come from another retrospective cohort study [31]. The authors identified 89 patients diagnosed with an HS flare from the University of Texas Southwestern Medical Center Inpatient Consult Registry. Of the sixty-four patients that satisfied inclusion requirements, 53.1% were female. The majority of patients (40.7%) had Hurley stage III disease, the median age was 39 years, and 79.7% of the patients were Black. SIRS criteria were met in 42.2% of patients upon admission, with the most prevalent being tachycardia (70.3%) and leukocytosis (51.6%). Lactate levels were elevated in 12.9% of the patients tested, and merely one patient reported fever (1.6%). A non-dermatologist misdiagnosed 51.7% of patients with sepsis when they arrived at the emergency room, and 82.8% of patients were given intravenous antibiotics for a median of three days due to suspected infection. Antibiotic resistance rates, needless testing, usage of antibiotics, and healthcare costs can all be reduced by acknowledging that patients experiencing HS flares can still fit the criteria for SIRS without developing sepsis.

### 3.6. Risk of Fournier’s Gangrene (FG) in HS Patients

Fournier’s gangrene (FG) is an uncommon life-threatening form of necrotizing fasciitis primarily involving the perineal, genital, and perianal regions [32]. It is characterized by rapid, progressive soft tissue necrosis caused by a polymicrobial infection, typically involving a combination of aerobic and anaerobic bacteria such as Group A *Streptococci, Staphylococcus aureus*, *E. Coli*, and *Pseudomonas aeruginosa* [33]. Predisposing factors include diabetes mellitus, hypertension, immunosuppression, alcohol misuse, and local trauma or infection, which facilitate bacterial entry and propagation [34,35]. Although it is uncommon for HS to directly advance to FG, physicians should exercise caution when treating patients who have severe HS including the perineal region. It is particularly important for patients with underlying risk factors such obesity, diabetes, or immunosuppression.

The simultaneous incidence of both HS and Fournier’s gangrene is remarkably rare, with only a few cases published thus far [12,13,14]. The frequency and nature of this relationship remain unknown, with the underlying processes that link the two disorders yet to be fully understood. According to the above-mentioned case reports, skin disintegration brought on by untreated severe HS in the anogenital region served as bacterial entry sites, resulting in a polymicrobial infection that eventually proceeded to Fournier’s gangrene. Furthermore, comorbid conditions, often present in HS patients, also play a potential role in the development of FG in those individuals. When treating FG, a tailored approach that includes surgical debridement and personalized antibiotic therapy is mandatory.

### 3.7. Risk of Osteomyelitis in HS Patients

Osteomyelitis is a condition that damages bone tissue and is brought on by an infection with pathogenic microbes. Early diagnosis, including bone collection for microbiological and pathological evaluation, is critical for effective care. Although rare, several cases of osteomyelitis associated with severe HS have been reported [15].

In the MRI performed, two patients presented with osteomyelitis of the sacrum and coccyx, and one with high T2-weighted signal intensity on the last sacral vertebra. Bone biopsy culture in each patient grew three distinct bacteria (*Streptococcus anginosus, Staphylococcus hominis, Streptococcus agalactiae*), proving a bacterial origin of osteomyelitis. All patients responded well to given antibiotic treatment. The authors concluded that though HS is not a bacterial disease, it can be a predisposing factor for osteomyelitis. What is more, they encourage performing an MRI to explore any severe gluteal and painful HS associated with increasing C-reactive protein (CRP). What is more, Mathew et al. have also described two cases of osteomyelitis linked to severe HS [16]. Both patients were treated with IV ertapenem for 6 weeks with satisfactory results.

### 3.8. Other

Notably, a study by Lee et al. delivered interesting data on cutaneous and extra-cutaneous infections associated with HS [36]. Patients with HS were more prone to developing infections than those with psoriasis; nonetheless, they were less so than patients with atopic dermatitis. Moreover, HS patients with comorbidities such as autoimmune disorders, acne, cardiometabolic risk factors, and mental health issues had a greater probability of developing an infection. Among reported infections, MSSA (methicillin-sensitive *Staphylococcus aureus*), MRSA (methicillin-resistant *Staphylococcus aureus*), strep infection, *Pseudomonas*, necrotizing fasciitis, bone infection, *Clostridium difficile* infection, pyelonephritis, septicemia, and antibiotic-resistant infections were statistically significant. What is more, association with infections leads to amplified mortality and costs of healthcare among HS patients.

## 4. Materials and Methods

**Figure 2:**
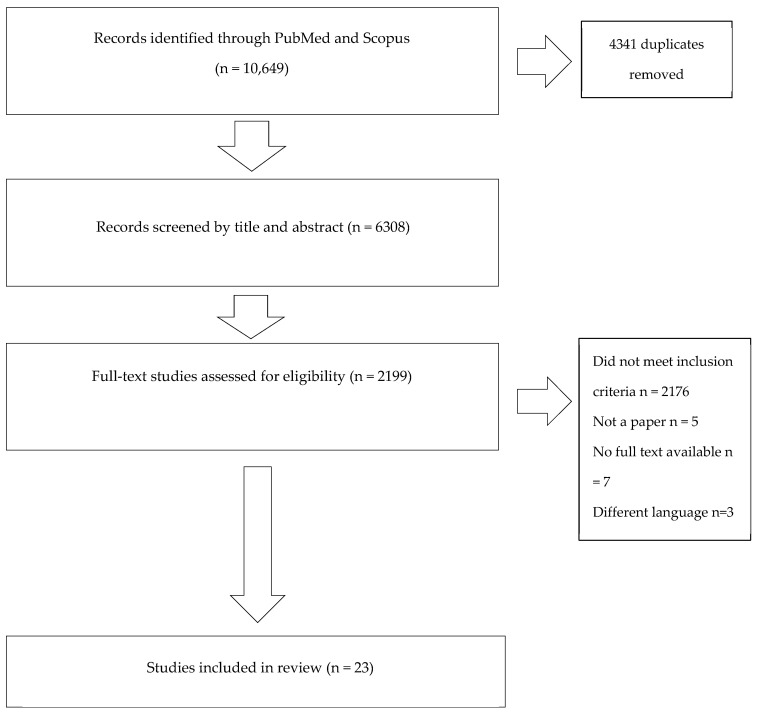
Flow diagram of narrative review.

The presented article is a narrative review. The PubMed and Scopus databases were searched for articles applicable to this review. The analysis reviewed the papers published by November 2024. An initial search was performed with terms “hidradenitis suppurativa” and “acne inversa”. This resulted in 5367 and 5282 results, respectively. Research was conducted with the main keywords “hidradenitis suppurativa” or “acne inversa” AND “bacterial infection” or “biofilm” or “bacteria” or “infection” or “Fournier’s Gangrene” or “cutaneous infection” or “sepsis”. The same search strategy was utilized on both databases. The inclusion criteria were as follows: studies reporting on bacterial infections in HS, published in the English language, with abstracts available, and randomized controlled trials, case–control studies, cross-sectional studies, and case reports were included. Duplicate articles, papers not related to the topic, and those written in languages other than English were excluded. The abstracts were screened by a single author (Z.Ś.) and, subsequently, the full texts were individually screened by two authors (Z.Ś. and W.B.-R.). The obtained articles were analyzed according to the inclusion criteria. The flow diagram of the narrative review is presented in Figure 2.

## 5. Conclusions

Patients with HS are susceptible to a broad spectrum of bacterial infections, ranging from localized purulent infections to severe systemic complications. What is more, bacterial infections can play a pivotal role in exacerbating HS activity. The polymicrobial nature of these infections, often involving biofilm-producing bacteria, perpetuates local inflammation and tissue damage, complicating disease management. Remarkably, comorbid conditions such as obesity and diabetes mellitus create a pro-inflammatory systemic milieu and alter the cutaneous microbiota, which exacerbates the severity of the disease and increases vulnerability to infections. Moreover, patients with uncontrolled or untreated HS are at a higher risk of developing infectious complications. These findings highlight the importance of a multidisciplinary approach that combines targeted antibacterial treatments with metabolic and immunological management to improve HS patient outcomes.

## Figures and Tables

**Table 1:** A summary of reported cases of bacterial infections in HS patients.

Authors (Year)	Diagnosis	Age (Years), Sex (Male/Female)	Symptoms	Outcome
Bessaleli, E, Scheinfeld, N (2018) [11]	*C. difficile* infection	63, female	Weakness, headaches, nausea, vomiting, diarrhea	Recovery after treatment with vancomycin
Khattar, G., Bou Sanayeh, E., Makram, M., Rabah, H., Abu Baker, S., Khan, S., & Hong, C. (2024) [12]	Fournier’s gangrene	42, female	Extensive wounds in the left axilla, perineum, lumbosacral and bilateral gluteal areas extending into the perineum	Hospitalization, death due to complications
Lau, A., Nguyen, N., Hui, A., Ong, J., & Salehpour, M. (2024) [13]		58, male	Necrotic tissue on the left groin, scrotum, and base of the penis	Hospitalization, recovery after surgical debridement and broad-spectrum IV antibiotic therapy
Magdaleno-Tapial, J., Valenzuela-Oñate, C., Martínez-Doménech, A., Sánchez-Carazo, J. L., & Pérez-Ferriols, A. (2019) [14]		38, male	Necrotic plaques on the lower abdomen and dorsum of the penis	Hospitalization, recovery after debridement and autologous partial skin grafts
Blaizot, R., Fernandez, P., Cogrel, O., Beylot-Barry, M., & Pham-Ledard, A. (2018) [15]	Osteomyelitis	53, male	Intense local pain, increased CRP, MRI: multiple fistulized abscesses and high T2-weighted signal intensity on the last sacral vertebra. Bone biopsy culture: *Streptococcus anginosus*	Hospitalization, recovery after treatment with clindamycin and levofloxacin
		30, male	Intense sacral pain and local suppuration without fever, increased CRP, MRI: a deep collection adjacent to osteomyelitis of the sacrum and coccyx. Bone biopsy culture: *Staphylococcus hominis*	Hospitalization, recovery after treatment with clindamycin and moxifloxacin
		52, male	Acute and intense localized pain and suppuration, raised CRP, MRI: osteomyelitis of the sacrum and coccyx. Bone biopsy culture: *Streptococcus agalactiae*	Hospitalization, recovery after treatment with amoxicillin, combined during the first 6 weeks with levofloxacin
Mathew, L., Goldenberg, S. D., Griffin, N., Ferguson, F. J., de la Roche, H. M., Hay, I., Lamb, R. C., & Rashidghamat, E. (2023) [16]		53, female	MRI: high STIR signal in the distal sacrum and coccyx	Hospitalization, recovery after treatment with IV ertapenem and oral linezolid, followed by excision (natal cleft down to the sacrum)
		54, male	MRI: high STIR signal changes in the coccyx	Hospitalization, recovery after treatment with IV ertapenem
Kathju, S.; Lasko, L.-A.; Stoodley, P. (2012) [6]	Biofilm formation	47, female	Painful, draining lesions in the buttock, perineum, and groin area	Hospitalization, recovery after wide excision

## Data Availability

No new data were created.
